# Impact of Home Quarantine on Physical Activity Among Older Adults Living at Home During the COVID-19 Pandemic: Qualitative Interview Study

**DOI:** 10.2196/19007

**Published:** 2020-05-07

**Authors:** Luc Goethals, Nathalie Barth, Jessica Guyot, David Hupin, Thomas Celarier, Bienvenu Bongue

**Affiliations:** 1 Autonomic Nervous System Research Laboratory University of Jean Monnet Saint-Etienne France; 2 Chaire Santé des Ainés University of Jean Monnet Saint-Etienne France; 3 Gerontopole Auvergne-Rhône-Alpes Saint-Etienne France; 4 Clinical physiology and Exercise Department University Hospital of Saint-Etienne Saint-Etienne France; 5 Department of Clinical Gerontology University Hospital of Saint-Etienne Saint-Etienne France; 6 Support and Education Technic Centre of Health Examination Centres (CETAF) Saint-Etienne France

**Keywords:** older adults, physical activity, COVID-19, social marketing

## Abstract

**Background:**

Older adults and those with pre-existing medical conditions are at risk of death from severe acute respiratory syndrome coronavirus 2 (SARS CoV-2). In this period of quarantine, one of the reasons for going out is physical activity. This issue is important, as the impact of a sedentary lifestyle might be lower for children and young adults, but is far more severe for older adults. Although older adults need to stay at home because they have a higher risk of coronavirus disease (COVID-19), they need to avoid a sedentary lifestyle. Physical activity is important for older adults, especially to maintain their level of independence, mental health, and well-being. Maintaining mobility in old age is necessary, as it may predict loss of independence in older adults.

**Objective:**

Our first objective was to evaluate the impact of this quarantine period on physical activity programs and on the physical and mental health of older adults. Our second objective was to discuss alternatives to physical activity programs that could be suggested for this population to avoid a sedentary lifestyle.

**Methods:**

We conducted a qualitative survey using semistructured interviews with professionals (managers in charge of physical activity programs for older adults and sports trainers who run these physical activity programs) from the French Federation of Physical Education and Voluntary Gymnastics (FFPEVG) and older adults participating in a physical activity program of the FFPEVG. We followed a common interview guide. For analysis, we carried out a thematic analysis of the interviews.

**Results:**

This study suggests that the COVID-19 epidemic has affected, before quarantine measures, the number of seniors attending group physical activity programs in the two study territories. In addition, despite the decline in their participation in group physical activities before the quarantine, older adults expressed the need to perform physical activity at home. There is a need to help older adults integrate simple and safe ways to stay physically active in a limited space. A national policy to support older adults for physical activity at home appears essential in this context.

**Conclusions:**

Given the results of our study, it seems necessary to globally communicate how important it is for older adults to maintain physical activity at home. We are concerned about the level of independence and mental health state of older adults after the end of quarantine if there is no appropriate campaign to promote physical activity among them at home.

## Introduction

After nearly 2 months of quarantine, France has approximately 132,000 people infected with coronavirus disease (COVID-19) and more than 25,000 deaths [1]. Older adults and those with pre-existing medical conditions are at risk of death from severe acute respiratory syndrome coronavirus 2 (SARS CoV-2) [2]. Studies agree that we are only at the beginning of an unprecedented health crisis affecting the population and especially older adults [3-5].

In this period of quarantine, one of the reasons for going out is physical activity [6]. However, this measure has been debated because it could lead to quarantine violation. This issue is important, as the impact of a sedentary lifestyle might be lower for children and young adults, but is far more severe for older adults. Although older adults need to stay at home because they have a higher risk of COVID-19 infection, they also need to avoid a sedentary lifestyle.

Physical activity is important for older adults, especially to maintain their level of independence [7], mental health, and well-being [8]. Physical inactivity among older adults is the fourth highest risk factor for mortality worldwide and a major contributor to disability [9]. Among people who do not engage in regular physical activity, the risk of functional decline is higher [10]. Maintaining mobility in old age is therefore necessary, as it may predict the loss of independence in older adults [11]. Insufficient physical activity during the quarantine period can therefore have deleterious effects on the mental and emotional health of older adults [12].

Our first objective was to evaluate the impact of this quarantine period on organizations conducting physical activity programs and on the physical and mental health of older adults. Our second objective was to discuss alternatives that could be suggested to this population to avoid a sedentary lifestyle.

## Methods

### Overview

We conducted a qualitative survey using semistructured interviews with professionals (managers in charge of physical activity programs for older adults and sports trainers who run these physical activity programs) from the French Federation of Physical Education and Voluntary Gymnastics (FFPEVG) and older adults participating in a physical activity program of the FFPEVG. We followed a common interview guide (Multimedia Appendices 1 and 2). 

The interviewees were sports trainers and older adults who were participating in the Social MArketing and Physical activity in Elderly (SMAPE) study [13]. The main objective of the SMAPE study was to determine whether a social marketing program based on the promotion of group balance workshops for people aged 60 years and over increases their attendance rate in sessions of “activities adapted physical skills.” This study was conducted in two French departments (Loire-42 and Haute-Loire-43). The FFPEVG organizes these group physical activity programs. The SMAPE study was suspended because of the quarantine implemented in France. 

### Data Collection

We asked the professionals about the impact of COVID-19 and quarantine on physical activity workshops and alternatives they could suggest. We also interviewed older adults about the importance they place on the physical activity before and during the quarantine and whether they were aware of other tools to continue physical activity at home. For analysis, we carried out a thematic analysis of the interviews.

## Results

For this study, 8 professionals (with different professional roles) and 6 older adults participated. The professionals responded unanimously that because of the COVID-19 epidemic, attendance at physical activity workshops has declined. Three sports trainers estimated the drop in attendance by “about 20%, but it’s hard to tell because it’s uneven across the different workshops.” A majority also informed us that some participants “no longer wanted to have close contact” with the other participants and “no longer wanted to touch the equipment.”

Older adults who were no longer attending the workshops preferred to abstain from these workshops to avoid contact with other participants and potentially contracting COVID-19. A majority of the professionals told us that there was also influence from the families of these older adults, who, despite interest in such group physical activity, did not want their close ones to risk exposing themselves to the virus.

Following the first announcement to contain COVID-19 propagation in France, the medical commission of the FFPEVG decided to cancel all physical activity workshops held in its clubs until further notice. Professionals expressed concern that shutting down these activities could isolate some seniors, many of whom live alone and often away from their families:

I'm in the countryside, my husband died two years ago, so I don't want to do much .

Marcelle, 80 years old

The professionals pointed out that there are alternative ways for older adults to perform physical activity at home. The FFPEGV proposed, for example, video clips on its website to help older adults exercise at home. However, none of the older adults were aware of the existence of online videos to encourage and assist them in performing physical activity at home:

No, I do not know of any tools.

Paulette, 84 years old

Moreover, respondents were not interested in using online videos to perform physical activity:

If we want to continue (sport), we do it like this [without video].

Renée, 91 years old

All the older adults were receptive to the importance of physical activity for their health:

I find it quite interesting, especially for me, I have a lot of osteoarthritides.

Renée, 91 years old

A majority continue to engage in physical activity at home:

I put C8, the channel (laughs) and I do the gym once in a while, and then some days I don't do it. It depends on how much time I have” .

Denise, 73 years old

Introducing physical activity into daily life requires the incorporation of a new culture into lifestyles that are provided, for example, in-group physical activity workshops:

I continue to do some things at home [based] on things I had seen and done there that I thought were good.

Renée, 91 years old

However, for some, quarantine plays a limiting role in the practice of physical activity. For some, it is because of the material conditions:

Walk? Yes a little bit...we live on the side of the national road, it's not easy for us either.

Josianne, 71 years old

Group physical activity creates social ties between people and encourages them to perform exercises. Therefore, being alone is an obstacle to performing physical activity:

On its own, it is less interesting.

Paulette, 84 years old

## Discussion

This study suggests that the COVID-19 epidemic has, before implementation of quarantine measures, affected the number of seniors attending group physical activity programs in the two study territories. This was mostly due to the fear of meeting potentially infected people.

Another result of our study is that despite the decline in older adults’ participation in group physical activities before the quarantine, they expressed the need to perform physical activity at home. Although quarantine is a measure to protect older adults from COVID-19, staying at home can lead to negative consequences such as reduced physical activity and sedentary behavior. It can also increase the risk of injury due to a lack of adapted equipment or poor knowledge of the physical exercises to perform. Moreover, social ties are essential to encourage older adults to perform physical activity [14]. Reduced social ties for older adults during quarantine could lead to a significant decrease in physical activity. In addition, loneliness could accelerate physical and cognitive decline in older adults [15].

The need for physical activity expressed by older adults raises the question of how older adults can be physically active in the current quarantine period. There is a need to help older adults integrate simple, safe ways to stay physically active in a limited space. A national policy to support older adults for physical activity at home appears essential in this context. In France, based on individuals’ initiatives, local structures have sent booklets of physical activity advice and exercises to older adults.

There are currently several online physical activity support systems. Some of them are very interesting, such as FFPEVG video clips [16], the website of the French Ministry of Sports [17], or the United Kingdom National Health Service guide [18] aiming to encourage older adults to perform physical activity at home. Our study suggests that older adults do not want to use these online tools. Given the results of our study, it seems necessary to globally communicate how important it is for older adults to maintain physical activity at home. Among older adults, there are cultural, sociological, and economic differences that need to be considered when developing targeted messages that echo a target audience’s existing views and practices, to produce more powerful persuasive effects. Behavioral segmentation can help better understand and target messages for high-risk subgroups [19] like older adults.

In conclusion, we are concerned about the level of independence and mental health status of older adults after the end of quarantine if there is no appropriate campaign to promote physical activity at home for them.

## Appendix

Multimedia Appendix 1 Interview guide for professionals.

Multimedia Appendix 2 Interview guide for older adults.

## Abbreviations

COVID-19: coronavirus disease
FFPEVG: French Federation of Physical Education and Voluntary Gymnastics
SARS CoV-2: severe acute respiratory syndrome coronavirus 2
SMAPE: Social MArketing and Physical activity in Elderly
