# Supplementary material for: Impact of Home Quarantine on Physical Activity Among Older Adults Living at Home During the COVID-19 Pandemic: Qualitative Interview Study
Source: JMIR Aging. 2020 May 7;3(1):e19007. doi: 10.2196/19007 (PMC7207013; doi:10.2196/19007)
Supplement: Multimedia Appendix 1 [file aging_v3i1e19007_app1.docx]

|  | **Interview guide**  **Project :**  **Need for a physical activity promotion strategy for older adults living at home during quarantine due to Covid-19**  Interview N° : N°  Date :  Place :  **1 Before the containment measures in France, what was the impact of COVID-19 on the attendance of your various workshops, particularly those on physical activity?**  **2. Did attendance decline?**  **3 If so, approximately how many people were absent?**  **4 If yes, did those who did not wish to participate explain why?**  **5 Did the elderly express any concerns?**  **6 Since the containment measures, what decisions have been made in terms of planning the workshops, particularly those on physical activity?** |
| --- | --- |
