# Supplementary material for: Impact of Home Quarantine on Physical Activity Among Older Adults Living at Home During the COVID-19 Pandemic: Qualitative Interview Study
Source: JMIR Aging. 2020 May 7;3(1):e19007. doi: 10.2196/19007 (PMC7207013; doi:10.2196/19007)
Supplement: Multimedia Appendix 2 [file aging_v3i1e19007_app2.docx]

|  | **Interview guide**  **Project :**  **Need for a physical activity promotion strategy for older adults living at home during quarantine due to Covid-19**  Interview N° : N°  Date :  Place :  **1. How important is it to you to recommend physical activity?**  **2. Before the containment measures in France, what types of physical activity workshops did you attend?**  **3. How often did you attend these workshops?**  **4. Did you stop attending these workshops even before the containment measures?**  **4.1. If so, can you explain why? Did you have any concerns?**  **4.2. If no, can you explain why?**  **5. Since the containment measures, do you practice physical activity at home?**  **5.1. If yes, can you explain what type of physical activity you do? Frequency? Are the materials available?**  **5.2. If no, can you explain why?**  **6. Are you aware of the FFEPGV videos animated by a sports coach to allow older people to practice physical activity at home?**  **6.1. If yes, how did you become aware of them? Are they useful to motivate you to practice physical activity at home?** |
| --- | --- |
